# Acute Exposure to Tris(1,3-dichloro-2-propyl) Phosphate (TDCIPP) Causes Hepatic Inflammation and Leads to Hepatotoxicity in Zebrafish

**DOI:** 10.1038/srep19045

**Published:** 2016-01-08

**Authors:** Chunsheng Liu, Guanyong Su, John P. Giesy, Robert J. Letcher, Guangyu Li, Ira Agrawal, Jing Li, Liqin Yu, Jianghua Wang, Zhiyuan Gong

**Affiliations:** 1 College of Fisheries, Huazhong Agricultural University, Wuhan 430070, China; 2 Collaborative Innovation Center for Efficient and Health Production of Fisheries in Hunan Province; 3 Department of Biological Sciences, National University of Singapore, Singapore 117543, Singapore; 4 Department of Chemistry, Carleton University, Ottawa, Ontario K1S 5B6, Canada; 5 State Key Laboratory of Pollution Control and Resource Reuse, School of the Environment, Nanjing University, Nanjing 210023, China; 6 Department of Veterinary Biomedical Sciences and Toxicology Centre, University of Saskatchewan, Saskatoon, Saskatchewan S7N 5B3, Canada; 7 Department of Zoology and Centre for Integrative Toxicology, Michigan State University, East Lansing, Michigan 48824, United States

## Abstract

Tris(1,3-dichloro-2-propyl) phosphate (TDCIPP) has been frequently detected in environmental media and has adverse health effect on wildlife and humans. It has been implicated to have hepatotoxicity, but its molecular mechanisms remain unclear. In the present study, adult male zebrafish were exposed to TDCIPP and global hepatic gene expression was examined by RNA-Seq and RT-qPCR in order to understand the molecular mechanisms of TDCIPP-induced hepatotoxicity. Our results indicated that TDCIPP exposure significantly up-regulated the expression of genes involved in endoplasmic reticulum stress and Toll-like receptor (TLR) pathway, implying an inflammatory response, which was supported by up-regulation of inflammation-related biomaker genes. Hepatic inflammation was further confirmed by histological observation of increase of infiltrated neutrophils and direct observation of liver recruitment of neutrophils labeled with Ds-Red fluorescent protein of *Tg*(*lysC:DsRed*) zebrafish upon TDCIPP exposure. To further characterize the hepatotoxicity of TDCIPP, the expression of hepatotoxicity biomarker genes, liver histopathology and morphology were examined. The exposure to TDCIPP significantly up-regulated the expression of several biomarker genes for hepatotoxicity (*gck*, *gsr* and *nqo1*) and caused hepatic vacuolization and apoptosis as well as increase of the liver size. Collectively, our results suggest that exposure to TDCIPP induces hepatic inflammation and leads to hepatotoxicity in zebrafish.

Tris(1,3-dichloro-2-propyl) phosphate (TDCIPP) has been used as flame retardants and plasticizers in various products (e.g. plastics, foams, textiles, varnishes, electronics equipment and furniture) for decades, and its annual production is estimated to be in the range of 4500 to 22,700 tons between 1998 and 2006 in the United States^1^. In recent years, TDCIPP has been increasingly used as the primary replacement of the phased-out flame retardant polybrominated diphenyl ether (PBDE)^1^.

Like PBDEs, TDCIPP is not chemically bonded to the related products and it is released to the environment easily^2^. TDCIPP is frequently detected in indoor air, dust, surface water, drinking water, influents, effluents, sediments, wildlife and human body^1^,^3^,^4^,^5^,^6^,^7^,^8^,^9^,^10^. For example, it has been documented that TDCIPP is present in more than 96% of the indoor dust samples in the United States and the concentrations range from <90 ng/g to 56,000 ng/g^11^. The concentrations of TDCIPP in surface water and effluent of sewage treatment plants in Germany and Norway have been reported to be up to 50 ng/L and 740 ng/L, respectively^1^,^3^. In the wildlife, TDCIPP has been detected at 36–140 μg/kg lipid weight in freshwater perches^10^. Recently, TDCIPP has also been detected in human milk and urine of office workers^10^,^12^,^13^.

Despite of its high volume of use and frequent detection in the environment, to date only limited information is available about the toxic effects of TDCIPP. For example, it has been reported that exposure to TDCIPP inhibits DNA synthesis and promotes neuron differentiation in PC12 cells^14^. In zebrafish, TDCIPP also causes developmental toxicity and endocrine disruption^15^,^16^,^17^,^18^,^19^,^20^. Exposure to TDCIPP in primary cultured avian hepatocytes also causes cytotoxicity with deregulation of genes involved in phase I and II metabolism, thyroid hormone pathway, lipid regulation and growth^21^. Furthermore, injection of TDCIPP to chicken eggs has resulted in a significant accumulation of TDCIPP in the liver and changes of expression of hepatic genes related to xenobiotic metabolism, thyroid hormone pathway and immune responses^22^,^23^. These studies suggest that liver is a target for TDCIPP exposure, however to support reliable risk assessment, underlying molecular mechanisms for hepatotoxicity need to be further explored.

The recently developed RNA-Seq technology provides a powerful tool to determine molecular mechanisms in organisms after chemical exposure, especially for emerging environmental pollutants with limited toxicological information since it allows a global examination of biological responses through gene expression. In this study, the effect of TDCIPP on zebrafish hepatic transcriptome was evaluated by RNA-Seq and we found an apparent inflammatory response based on TDCIPP-induced transcriptomic changes. The inflammatory response was confirmed by up-regulation of some biomarker genes and migration of neutrophils to the liver following TDCIPP treatment. Finally, the hepatotoxicity of TDCIPP was further characterized by measuring the expression of biomarker genes for hepatotoxicity and the change of liver histology and morphology.

## Materials and Methods

### Animals and Chemical Exposure

TDCIPP was purchased from Sigma (St. Louis, MO, USA) and dissolved in dimethyl sulfoxide (DMSO) as a stock solution. Briefly, wild-type male zebrafish (Singapore strain, 5 months old) were acclimated for 2 weeks in our aquarium at 28 °C, with a 14:10 light/dark cycle. Zebrafish were then exposed to 0.01% DMSO (vehicle control) or 1 mg/L TDCIPP for 4 days in 15 L glass tanks with 10 L exposure solution, and half of the water with the same chemical of the same concentration was daily renewed. During semi-static exposure period, water pH (7.0–7.3), hardness (3-4 dGH) and dissolved oxygen (6.4–6.8 mg/L) were routinely monitored. The exposure concentrations were selected based on the information from a previous study^16^, where exposure to 1 mg/L TDCIPP caused reproductive toxicity and endocrine disruption in adult zebrafish. There were two replicated tanks for each group and each tank contained 12 fish. No mortality was observed in any of the treatment and control groups during the exposure period. After the exposure, the fish were anesthetized and livers were collected for RNA extraction, RNA-Seq and Western blotting analyses. All experimental procedures in this study were carried out following the approved protocol by Institutional Animal Care and Use Committee of National University of Singapore (Protocol 079/07). All experiments of this study were performed in accordance with relevant guidelines and regulations in Singapore.

In order to confirm our findings in RNA-Seq, the second set of TDCCP exposure experiments, including time- and dose-dependent exposure experiments, were conducted. Wild-type male zebrafish (5 months old) were acclimated for 2 weeks, followed by exposure to 0.01% DMSO (vehicle control) or different concentrations of TDCIPP (0.1, 0.3 or 1 mg/L) as above described. There were three replicated tanks for each group, and each tank contained 12 fish. For 1 mg/L exposure group, fish were sampled after 1, 2 and 4 days of exposure; for 0.1 and 0.3 mg/L exposure groups, fish were sampled only after 4 days of exposure. Liver tissues were preserved in TRIzol reagent (Invitrogen, New Jersey, NJ, USA) for RT-qPCR validation or collected for histological examination. No mortality was observed in any of the treatment and control groups during the exposure period. For dose-dependent exposure experiment, one mL exposure solution from each tank was collected before and after renewal of the water solutions on the last day of exposure, and concentrations of TDCIPP were quantified.

### Quantification of TDCIPP in Exposure Solutions

Exposure solutions were sampled before and after renewal of the water solutions on the last day of exposure, and concentrations of TDCIPP were quantified according to previously published protocols^24^,^25^. Briefly, exposure solutions were filtered through 0.22 μm membrane filters and then diluted using Milli-Q water. Water samples were analyzed using a Waters ACQUITY UPLC^®^ I-Class system (UHPLC) coupled to Waters^®^ Xevo^TM^ TQ-S mass spectrometer (TQ-S/MS) (Milford, MA, USA) using electrospray ionization (ESI(+)) in the multiple reaction monitoring (MRM) mode. LC separation was performed on a Cortecs^TM^ UHPLC C18 column (2.1 mm × 50 mm, 1.6 μm particle size) (Waters, Mississauga, ON, Canada). Mobile phases for LC were water (A) and methanol (B), and flow rate was set to 0.5 mL/min. Gradient was as follows: 0 min, 5% B; 0−5 min, 95% B (linear); hold for 1 min; 6−6.1 min, 5% B (linear) and hold for 4.9 min. The capillary voltage was 0.5 KV. The source and desolvation temperatures were 150 and 600 °C, respectively. The desolvation and cone gas flow rates were 800 and 150 L/h, respectively. Method limits of quantification were 0.01 ng TDCIPP /mL water. Triplicates of beakers were conducted for each concentration.

### RNA Isolation and Sequencing

Total RNA was isolated using TRIzol reagent and treated with DNase I (Invitrogen, New Jersey, NJ, USA) to remove genomic DNA contamination. RNA concentrations, ratios of 28S/18S and RNA integrity were determined using Agilent Bioanalyser 2100 (Agilent Technologies, Inc., Santa Clara, CA, USA). RNA concentrations ranged from 1 to 2 μg/μL, and ratios of 28S/18S ranged from 1.85 to 2.05. Six livers were pooled to generate one biological replicate for RNA preparation, and two biological replicates from two individual tanks were included for each treatment group. Magnetic beads with Oligo(dT) were used to isolate poly A + RNA, which was then fragmented into short fragments in fragmentation solution. cDNA was synthesized using the mRNA fragments as templates, and short fragments were purified and ligated with adapters. After agarose gel electrophoresis, suitable fragments were selected for PCR amplification and sequenced as 2 × 90 bp paired-end reads on Illumina HiSeq^TM^ 2000 sequencer (Illumina, San Diego, CA, USA). RNA sequencing was performed by BGI Tech Solutions (Hong Kong) Co., Limited using the Illumina’s Solexa platform.

### Sequence Tag Preprocessing, Mapping and Statistical Analyses

The original image data were transferred into sequence data and saved as FASTQ files. The quality control of alignment was performed to determine if resequencing was necessary. Briefly, the raw reads were cleaned by removing reads with adaptors, low sequence quality (>30%) or high-proportion unknown bases (>5%) in a read. The clean data were then mapped to the zebrafish Reference Sequence database (http://www.ncbi.nlm.nih.gov/RefSeq) using the SOAPaligner/SOAP2 software, with allowance of maximum 5 nucleotide mismatches. Finally, the alignment data was utilized to calculate the distribution of gene coverage. Gene expression level was normalized as RPKM (reads per kilobase transcriptome per million mapped reads). In this study, we selected differentially expressed genes based on fold change >2 (statistical power >0.8) and *P* value < 0.05.

### Gene Ontology and Pathway Analyses

Gene ontology and pathway analyses were conducted using DAVID (The Database for Annotation, Visualization and Integrated Discovery) with the total zebrafish genome information as the background^26^. Gene Ontology Fat and KEGG-pathway categories were used in this study and *P* value (modified Fisher’s exact t-test) cut-off was set at 0.05.

### Reverse Transcription-Quantitative Polymerase Chain Reaction (RT-qPCR)

Total RNA was isolated using TRIzol reagent and treated with DNase I to remove genomic DNA contamination as previously described^27^. The synthesis of first-strand cDNA and RT-qPCR were performed by use of Maxima^®^ First Strand cDNA Synthesis kit (Fermentas, St Leon-Rot, Germany) and SYBR^®^ Green PCR kit (Toyobo, Osaka, Japan), respectively according to the manufacturer’s instructions. PCR primers were designed using Primer 3 software (http://frodo.wi.mit.edu/as) (Table S1, see Supporting Information), and glyceraldehyde-3-phosphate dehydrogenase (*gapdh*), whose cycle threshold (Ct) values were not changed upon TDCIPP exposure in this study (Figure S1), was used as an internal reference. The mRNA levels were expressed as fold change using the 2^-ΔΔCt^ method. There were 3 replicated tanks for each concentration, and three fish from each tank were used and thus totally 9 fish were analyzed in each treatment.

### Western Blotting Analyses

Western blotting analyses were performed as previously described with some modifications^28^. After exposure, liver tissues were sampled and homogenized. Homogenates were centrifuged for 5 min at 4 °C and protein contents were determined using commercial BCA (bicinchoninic acid) kit from Sigma (St. Louis, MO, USA). Equal quantity of proteins (50 μg) from control and exposure groups were denatured, electrophoresed and transferred onto polyvinylidene difluoride (PVDF) membranes. Membranes were cut into stripes (Figure S2) and blocked by 5% non-fat dry milk for 1 h. The blots were probed with primary antibodies from Cell Signaling Technology, Inc. (MA, USA) for 12 h at 4 °C, followed by three washes and incubation with corresponding secondary antibodies (Cell Signaling Technology, Inc., MA, USA) for 30 min at room temperature. ECL^TM^ reaction solution was prepared according to the manufacturer’s instructions (PerkinElmer Inc., SC, USA). Membranes were washed three times, and exposed to Kodak film for two min at room temperature. Films were developed and Grp78, Chop, Fos and Il6 were detected by chemiluminescence.

### Zebrafish Larva Assays

Two transgenic zebrafish lines were used in the present study: *Tg*(*lysC:DsRed*)^29^ and *Tg*(*fabp10a:DsRed; elaA:egfp*)^30^, named *Tg*(*fabp10a:DsRed)* in the following text. *Tg*(*lysC:DsRed*) line has red fluorescent protein (DsRed) expression in neutrophils and it is feasible to observe recruitment of neutrophils in the liver^31^ and other organs^29^. *Tg*(*fabp10a:DsRed)* line has liver-specific DsRed expression under the gene *fabp10a* promoter and allows an easy measurement of liver size^30^. It has been confirmed to be useful in screening hepatotoxin (e.g., acetaminophen, aspirin, isoniazid and phenylbutazone) which can induce hepatic damages, oxidative stress and cellular necrosis^32^. The embryos from the two transgenic lines were collected and cultured in egg water as described in a previous study^33^. At 96 hour postfertilization (hpf), larvae from the two transgenic lines were exposed to 0.01% DMSO or different concentrations of TDCIPP (0.1, 0.3 or 1 mg/L) in 6-well plates. Half of the exposure media was daily replaced. There were three replicated wells for each exposure concentration at each sampling time point, and each well contained 10 larvae. Both TDCIPP and control groups received 0.01% DMSO. For 1 mg/L exposure group, fish were sampled after 1, 2 and 4 days of exposure; for 0.1 and 0.3 mg/L exposure groups, fish were sampled only after 4 days of exposure. The number of neutrophils in the liver area in *Tg*(*lysC:DsRed*) larvae was determined using Carl Zeiss Axiovert 200M fluorescent and Carl Zeiss LSM 510 Meta fluorescent microscopes. Liver size was measured for *Tg*(*fabp10a:DsRed*) larvae using ImageJ software (http://rsbweb.nih.gov/ij/) based on 2D liver images as previously described^32^.

### Histological Examination

After 4 days of exposure, the liver tissues from males were sampled for histological examination as previously described^34^. Briefly, the livers were fixed in Bouin’s solution and dehydrated in ethanol. The samples were then embedded in paraffin wax, sectioned at 5 μm and stained with haematoxylin and eosin.

### Statistical Analysis

Statistical analyses of the data for the number of neutrophils in the liver, liver size and gene expression were conducted using Kyplot Demo 3.0 software (Tokyo, Japan). Normality and homogeneity of data were evaluated by the Kolmogorov-Smirnow and Levene’s tests, respectively. ANOVA (one-way analysis of variance) was adopted to determine significant differences between the control and TDCIPP exposure groups. A level of significance for type I error was set at *P* value < 0.05.

## Results

### Measured Concentrations of TDCIPP in Exposure Solutions

The nominal concentrations of TDCIPP in the exposure solutions were 0.1, 0.3 and 1 mg TDCPP/L. The analytical measured and actual TDCIPP concentrations in the same three solutions were 0.12 ± 0.00, 0.41 ± 0.01 and 1.13 ± 0.05 mg/L before water renewing, and 0.12 ± 0.00, 0.43 ± 0.02 and 1.17 ± 0.02 mg/L after water renewing, respectively (Figure S3, see Supporting Information).

### Transcriptomic Responses to TDCIPP in Adult Zebrafish Liver

No mortalities were observed in any of the treatment groups during exposure period. To analyse transcriptomic responses in the liver following acute TDCIPP exposure, four RNA libraries were constructed for RNA-Seq: two replicates from the TDCIPP treatment group (1 mg/L, 4 days) and two replicates from the 0.01% DMSO vehicle treatment group. Representative images for the composition and quality distribution of bases are showed in Figures S4 and S5 (see Supporting Information), where the T and C curves were in accordance with the A and G curves, respectively (Figure S4, see Supporting Information). The percentage of the bases with low quality (<20) was very low in all the samples tested (Figure S5, see Supporting Information), indicating good-quality of sequencing data without the need of resequencing. After filtering out reads with adaptors, low sequence quality (>30%) or high-proportion unknown bases (>5%), over 50 million clean reads were obtained from each library (Table S2) and over 70% of these reads (or at least 35 million from each library) were mappable to the zebrafish Reference Sequence database (http://www.ncbi.nlm.nih.gov/RefSeq), representing a total of 16,631 genes (Table S2). Using fold change >2 and *P* value < 0.05 as selection criteria, 583 differentially expressed genes (306 up-regulated and 277 down-regulated) were identified between control and TDCIPP groups (Figure S6 and excel data, see Supporting Information). Furthermore, using relative (log 2) RPKM values of the 583 differentially expressed genes, regression analysis (standard line assay) was conducted among the four RNA-Seq groups. As shown in Figure S7 (see Supporting Information), the adjusted correlation coefficients (R^2^) were very high within the same treatment group (DMSO control, 0.9181; TDCIPP, 0.9612), but low between the two groups (0.6155–0.6842), indicating good repeatability and reliability of our data.

The up- and down-regulated transcripts were further subjected to Gene Ontology (GO) and KEGG pathway analyses. The most significantly enriched GO term for biological process was Innate immunity response, where five toll-like receptor (*tlr*) genes were included (Table 1). Other relevant enriched terms in the biological process include Defense response, Response to inorganic substance, Transmembrane transport, Metal ion transport, Cation transport, Ion transport, Response to xenobotic stimulus, and Response to virus (Table 1). Only one enriched GO term with 14 deregulated genes, endoplasmic reticulum (ER), was observed for cellular component (Table 1). The enriched terms for molecular function included nucletidyltransferase activity, oxidoreductase activity (acting on the CH-NH2 group of donors, oxygen as receptor) and dipeptidyl-peptidase activity. In KEGG pathway analysis, two enriched terms, toll-like receptor signaling pathway and steroid hormone biosynthesis, were obtained (Table 1). For toll-like receptor signaling pathway, 6 genes were involved, including 5 up-regulated and 1 down-regulated genes (Table 1).

### Time- and Dose-Dependent Response of Biomarker Genes Involved in ER Stress and Inflammation

To validate our findings in RNA-Seq, time- and dose-dependent exposure experiment was conducted and expression of 19 selected genes involved in Innate immune response (GO)/Toll-like receptor signaling pathway (KEGG), ER response and Inflammation response, were determined by RT-qPCR (Table 2 and Table 3). TDCIPP exposure caused a time-dependent up-regulation of genes enriched in Innate immunity response/Toll-like receptor signaling pathway. While the expression of these genes (*tlr18*, *tlr8a*, *tlr8b*, *tlr20a*, *tlr9*, *fos*, *stat1b* and *irf7*) was not significantly altered after 1 day of TDCIPP treatment, their expression was all significantly up-regulated by 2 days of exposure and by 4 days of TDCIPP treatment. The two ER stress biomarker genes, *grp78* (glucose-regulated protein 78) and *chop* (CCAAT/enhancer-binding protein-homologous protein), also showed similar up-regulation by TDCIPP, with 3.2- and 9.9-fold up-regulation for *grp78* and 1.9- and 10.6-fold up-regulation for *chop* after 2 and 4 days of exposure, respectively. Finally, abundances of some marker genes for inflammation were also examined after TDCIPP exposure for 1, 2 and 4 days. At least seven of them (*il1b*, *il6, il10, il12a, il13, il15* and *il26*) also showed time-dependent increase following TDCIPP. The highest up-regulated genes were two interleukin genes, *il13* and *il26*, with over 23 fold of increase of expression after 4 days of TDCIPP exposure. Exposure to lower concentrations of TDCIPP (0.1 or 0.3 mg/L) for 4 days only up-regulated the expression of *stat1b*, *irf7*, *grp78*, *il13*, *il22* and *il26*, while the expression of other genes was not significantly changed.

**Figure 1:**
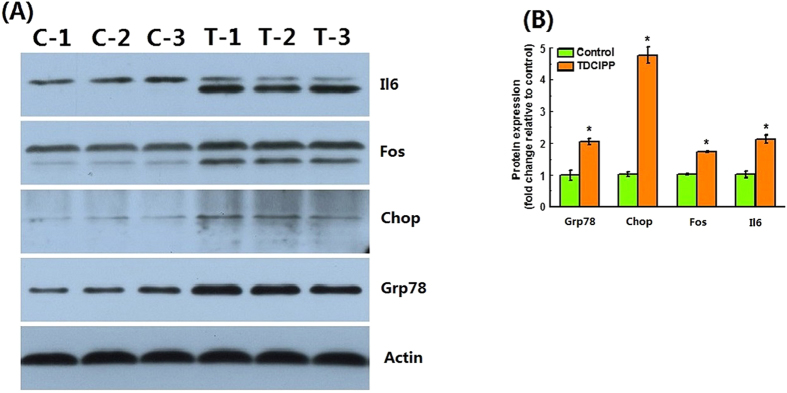
Effects on expressions of four proteins selected (Grp78, Chop, Fos and Il6) in response to 1 mg/L TDCIPP. (**A**) Western blots of four proteins selected from control and TDCIPP groups; (**B**) Quantification of the relative expressions of four proteins selected in control and treatment groups. **C**: control group; T: treatment group. Values represent mean ± SEM (n = 3). Asterisks indicate significant differences from matched control samples (*P* < 0.05).

To further confirm the up-regulation of some of these genes at protein level, four proteins from Toll-like receptor signaling pathway (Fos), ER stress (Grp78 and Chop) and Inflammation response (Il6) were selected for Western blot analysis because of the availability of their antibodies. Their expression in liver of male zebrafish exposed to 0 or 1 mg TDCIPP/L was examined. As shown in Fig. 1, exposure to 1 mg TDCIPP/L for 4 days significantly up-regulated expressions of Grp78, Chop, Fos and Il6 by 2.05, 4.77, 1.71 and 2.13 fold, respectively.

### Up-regulation of Hepatotoxicity Biomarker Genes by TDCIPP

**Figure 2:**
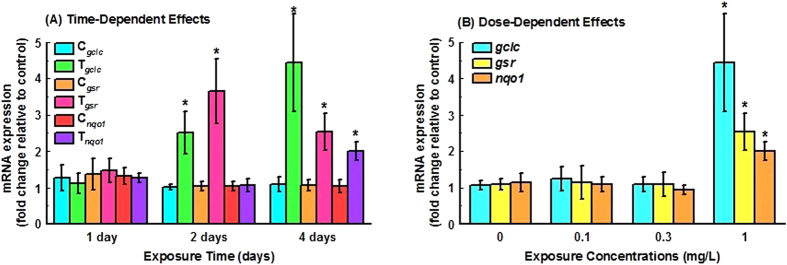
Dose- (A) and time-dependent (B) effects on the expression of selected hepatotoxicity biomarker genes (*gclc*, *gsr* and *nqo1*) in response to TDCIPP.(C): control group; T: treatment group. Values represent mean ± SEM (n = 9). Asterisks indicate significant differences from matched control samples (*P* < 0.05).

To further confirm the hepatotoxicity caused by TCDPP exposure, a panel of 14 hepatotoxicity biomarker genes included in “generalized hepatotoxicity” based on Qiagen Hepatotoxicity RT2 Profiler PCR array (http://www.qiagen.com/) were selected for RT-qPCR analysis. We found that exposure to 1 mg/L TDCIPP for 2 or 4 days up-regulated the expression of three of these biomarker genes, *gclc* (glutamate-cysteine ligase catalytic subunit), *gsr* (glutathione reductase) and *nqo1* (NAD(P)H dehydrogenase [quinone] 1 isoform 1) (Fig. 2A), while expression of other genes (*krt8, plazg12a, hmox1, krt18, gadd45ab, cryl1, ccng1, casp3b, casp3a, apex1 and aldoaa*) was not significantly changed (data not shown). Furthermore, out of the 14 genes selected, thirteen genes were detected in RNA-seq, and only *plazg12a* was not detected possibly due to low expression abundance. The fold changes of the 13 genes were consistent in qRT-PCR with those in RNA-seq although up-regulation of *gclc* (1.52) and *nqo1* (1.77) was not statistically significant in RNA-Seq data and thus the two genes were not initially selected by out cutoff criteria for differentially expressed genes. No significant changes were observed after exposure to lower concentrations of TDCIPP (0.1 or 0.3 mg/L) for 4 days (Fig. 2B).

### Validation of Inflammatory Response and Hepatotoxicity by Histological Examination and Transgenic Zebrafish Larvae

**Figure 3:**
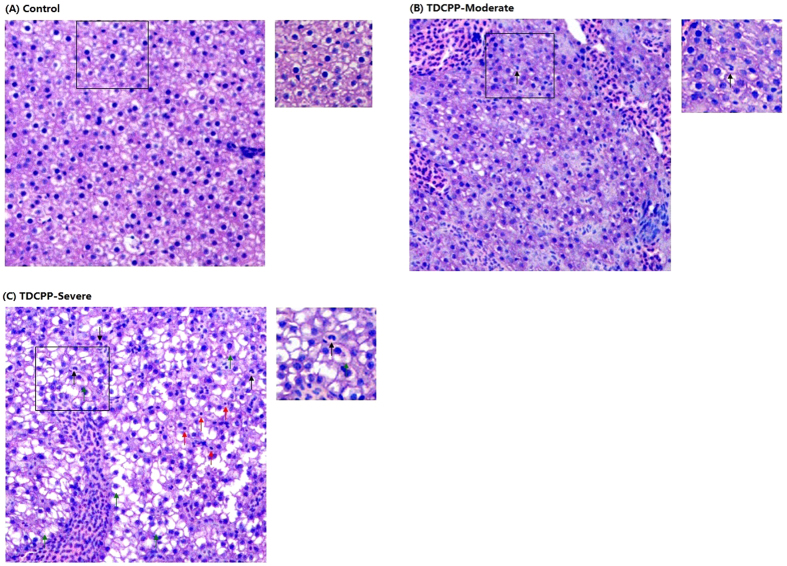
Changes of liver histology of male zebrafish after exposure to 1 mg/L TDCIPP for 4 days. Liver sections were stained by hematoxylin and eosin. (**A**) representative liver section from the DMSO vehicle control group. (**B,C**) Two representative liver sections from two individual fish of the same TDCIPP group with a moderate effect (**B**) and a severe effect (**C**). The left images have a magnification of 200X and the right images have a magnification of 400X. Several features are exampled by arrows of different colors: neutrophils (black); apoptosis (red); hepatic vacuolization (green).

As RNA-Seq data indicated a potential inflammatory response caused by TDCIPP exposure, histological examination was also carried out for the effects of TDCIPP on liver cells. In this experiment, adult zebrafish were exposed to 1 mg/L TDCIPP for 4 days. Histological examination revealed that the TDCIPP exposure caused an increase of infiltrated neutrophils, hepatic vacuolization and apoptosis (Fig. 3). Examples of moderately and severely affected liver sections are shown in Fig. 3B,C, respectively.

**Figure 4:**
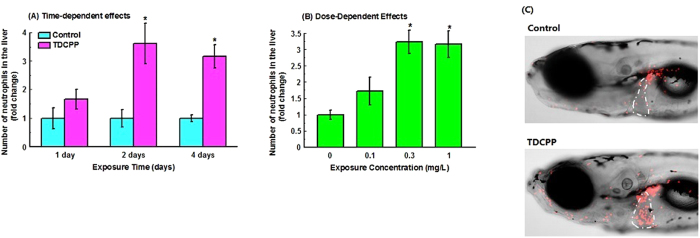
Dose- (A) and time-dependent (B) increase of neutrophils in the livers of *Tg*(*lysC:dDsRed*) zebrafish larvae in response to TDCIPP exposure.Images were captured with a digital camera attached to a Carl Zeiss LSM 510 Meta fluorescent microscope. (**C**) Representative images from control group and 4-day TDCIPP (1 mg/L) exposure group. The livers are outlined with white lines. Values represent mean ± SEM. Significant difference between the two groups were observed: *P* < 0.05.

To further confirm the inflammatory response induced by the TDCIPP exposure, *Tg*(*lysC:DsRed*) zebrafish larvae, in which neutrophils were labelled by DsRed expression, were treated with different concentrations of TDCIPP from 96 hpf. No mortalities were observed in any of the treatment groups during the exposure period. Exposure to TDCIPP caused time- and dose-dependent increases in infiltration of neutrophils in the liver, while no significant infiltration was observed in other internal organs (Fig. 4A–C). In addition, slight increase of neutrophils was observed in the ventral region of the head and near the mouth (Fig. 4A–C).

**Figure 5:**
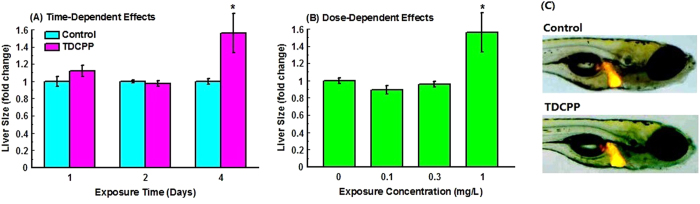
Dose- (A) and time-dependent (B) increase of liver size in *Tg*(*fabp10a:DsRed*) zebrafish larvae after exposure to TDCIPP.Images were captured with a digital camera attached to a Carl Zeiss LSM 510 Meta fluorescent microscope. Liver sizes were measured based on the 2D image using the ImageJ software. (**C**) Representative images from control group and 4-day TDCIPP (1 mg/L) exposure group Values represent mean ± SEM. Significant difference between the two groups were observed: *P* < 0.05.

Transgenic zebrafish *Tg*(*fabp10a:DsRed*) was also used to investigate the effect of TDCIPP and this transgenic line has been previously suggested to be a useful model to evaluate the hepatotoxicity of chemicals^32^. In the present study, no mortalities were observed in any of the treatment groups during TCDPP exposure; the liver size was significantly increased in a time- and dose-dependent manner compared with that of the control (Fig. 5A–C).

## Discussion

It has been recently demonstrated that TDCIPP is significantly accumulated in the livers of chicken after exposure and the expression of hepatic genes included in xenobiotic metabolism, thyroid hormone pathway, lipid regulation and immune responses are deregulated^21^,^22^,^23^, thus implying an apparent hepatotoxicity. However, these studies only examined responses of certain genes involved in a few pathways, and the information provided is rather limited. To devise a reliable risk assessment, further study is needed for a more comprehensive examination of gene responses by using “omic” technologies. In the present study, we have evaluated the effects of TDCIPP on hepatic transcriptome by RNA-Seq in zebrafish. Our data suggest that acute exposure to TDCIPP significantly changes the expression of genes involved in TLR pathway and ER stress in a dose- and time-dependent manner. More interestingly, TDCIPP also leads to the up-regulation of some biomaker genes for inflammation, which has been confirmed by increased neutrophil infiltration in the adult liver and rapid migration of neutrophils to the larval liver. Finally, TDCIPP exposure up-regulates the expression of several biomarker genes for hepatotoxicity (e.g. *gclc*, *gsr* and *nqo1*) and causes hepatocyte vacuolization, apoptosis in liver cells and increase of liver size, further proving the hepatotoxicity of TCDPP.

In this study, transcriptional effect of TDCIPP on the liver has been first evaluated in zebrafish. GO and KEGG pathway analyses indicate that a number of biological processes and pathways are significantly altered. The most significantly enriched GO term for cellular component is Innate immunity response, where five *tlr* genes are included. KEGG pathway analysis has further confirmed that TDCIPP exposure significantly changes the expression of genes in TLR signaling pathway, including *tlr8a*, *tlr8b*, *tlr9*, *ap-1*, *stat1b* and *irf7*. In mammalian livers, TLR genes are expressed in Kupffer cells, hepatocytes, stellate cells, biliary epithelial cells, sinusoidal endothelial cells, dendritic cells and other types of immune cells^35^,^36^. The protein products of these TLR genes are pattern recognition receptors and, once activated, they can interact with a common adaptor, MyD88 (myeloid differentiation factor 88), to activate nuclear transcription factors such as NF-κB, AP-1 and IRFs, and to cause the initiation of innate immunity^36^. In addition, activated IRFs can interact with interferons (IFNs) to activate STAT1, and cause corresponding inflammatory responses^37^. In this study, exposure to TDCIPP significantly up-regulates the expression of *tlr8b*, *tlr9*, *ap-1*, *irf7* and *stat1b* in a dose- and time-dependent manner. Consistent with this, we have found an up-regulation of eight cytokine genes/proteins in the liver after TDCIPP exposure. Cytokines are a family of secreted and regulatory molecules with molecular masses ranging from 10 to 50 kDa^38^. Besides classical responses and interactions between immune and neuroendocrine systems, cytokines play a fundamental role in inflammation^38^, and these genes are considered as main biomarkers of inflammation in fish^39^. In addition, an increase of neutrophils in the liver of TDCIPP-treated adult fish and *Tg(lyzC:DsRed)* zebrafish fry are also observed. Although neutrophil infiltration might be also due to liver damage (e.g., fatty liver) induced by TDCIPP, the up-regulation of biomarker genes for inflammation strongly suggests that inflammatory responses do occur.

Recently, it has been reported that some TLRs, such as TLR9, are exclusively sequestered in the ER in unstimulated cells and traffic to endolysosomes upon ligand stimulation^40^. Moreover, ER stress can directly induce TLRs and synergise with TLRs to cause inflammatory responses or/and related diseases in the liver^35^,^41^,^42^,^43^,^44^,^45^. In this study, ER is also a significantly enriched GO term, where the expression of 14 genes is significantly altered during TDCIPP exposure. For example, the expression of *pdia4*, *hyou1*, *hsp90b1*, *hspa8* and *pdip5* is significantly down-regulated after TDCIPP exposure. The protein products of these genes are ER chaperones^46^,^47^,^48^,^49^,^50^ and the down-regulation of these genes is the evidence for decreased ER function due to stress. Similarly, *slc35b1* (solute carrier family 35 member B1) is responsible for sugar transport in ER^51^ and exposure to 1 mg/L TDCIPP for 4 days significantly down-regulates its expression. Thus, the down-regulation of *slc35b1* may be another evidence for the occurrence of ER stress. In addition, treatment with TDCIPP significantly up-regulates the expression of *sgk1;* this is reminiscent of a previous report that ER stress in PC12 cells induced by overexpression of β-amyloid precursor protein are also accompanied by the up-regulation of sgk1 expression^52^. Accumulated evidence indicates that when ER stress occurs, cells could initiate an adaptive response called UPR (unfolded protein response) to maintain homeostasis of ER function, such as increase in the expression *grp78* and *chop*^53^,^54^,^55^. Protein coded by *grp78* works as a sensor for accumulation of unfolded proteins; once activated it will cause UPR^54^. Chop plays a key role in the downstream of UPR, and works as a chaperone of other proteins (e.g., cleaved Atf6α and Xbp-1) to mediate inflammatory response and cellular apoptosis^54^. In this study, TDCIPP causes a time-dependent up-regulation of the two genes. Treatment with 1 mg/L TDCIPP for 4 days also increases protein expressions of Grp78 and Chop. However, the expressions of *grp78* and *chop* were not detected in RNA-seq, which might be due to their low abundances of expression. Collectively, our observations suggest that ER stress occurs in zebrafish liver upon TDCIPP exposure, which might be a main reason for the up-regulation of genes included in the Toll-like receptor pathway.

Finally, we have characterized the hepatotoxicity of TDCIPP by measuring the expression of related genes and the change of liver histology and morphology. In this study, a panel of hepatotoxicity biomarker genes based on Qiagen Hepatotoxicity RT2 Profiler PCR array (http://www.qiagen.com/) were selected for RT-qPCR analysis, and expressions of three biomarker genes including *gclc*, *gsr* and *nqo1* were significantly up-regulated. The three genes encode antioxidant and detoxifying enzymes in liver^56^, and up-regulation of their expression are usually used as biomarker of hepatotoxicity^56^. Therefore, our results confirm that TDCIPP has potential to induce hepatotoxicity in zebrafish. Histological evidence indicates that exposure to TDCIPP causes hepatocyte apoptosis and vacuolization. Furthermore, we have also used *Tg*(*fabp10a:DsRed*) transgenic zebrafish to further evaluate the effect of TDCIPP on liver size. This transgenic line has RFP expression in the liver and it is feasible to measure the size of liver. We have found that treatment with 1 mg/L TDCIPP for 4 days significantly increases liver size. Consistent with this, a previous study also suggested that hepatic inflammation was accompanied by enlarged hepatosomatic index in fish exposed to pollutants^57^.

Here it should be noted that inflammatory response might be also caused by liver injury and apoptosis induced by TDCIPP, therefore further studies are needed to explore these possibilities. In addition, the concentrations (0.1, 0.3 and 1 mg/L) of TDCIPP that fish have been exposed in this study is several orders of magnitude greater than those reported in the environment, but our study represents an acute toxic experiment. Due to increased use and frequent detection of TDCIPP, further studies to evaluate the potential toxic effects of TDCIPP by chronic exposure with low concentrations are required.

## Additional Information

**How to cite this article**: Liu, C. *et al*. Acute Exposure to Tris(1,3-dichloro-2-propyl) Phosphate (TDCIPP) Causes Hepatic Inflammation and Leads to Hepatotoxicity in Zebrafish. *Sci. Rep*. **6**, 19045; doi: 10.1038/srep19045 (2016).

## Supplementary Material

Supplementary Information

Supplementary table S1

## Figures and Tables

**Table 1:** Enriched GO terms and pathways in response to TDCIPP exposure in zebrafish liver (*P* < 0.05).

	Terms	Count	*P* value	Fold enrichment	Genes
Gene Ontology					
Biological Process	Innate immune response	5	5.39E-3	6.92	*tlr18, tlr8a, tlr8b, tlr20a, tlr9*
	Defense response	6	1.32E-2	4.22	*tlr18, zgc:194626, tlr8a, tlr8b, tlr20a, tlr9*
	Response to inorganic substance	5	1.45E-2	5.23	*zgc:174006, mt2, per1a, mxb, slc40a1*
	Transmembrane transport	17	1.45E-2	1.92	*slc22a18, slc5a1, zgc:175280, aqp7, si:dkey-5g7.3, slc16a9a, slc35b1, zgc:77158, slc26a5, slc25a33, slc8a2a, slc13a1, slc13a2, slc25a32a, slc25a39, slc30a10, si:dkey-246g23.4*
	Metal ion transport	12	1.94E-2	2.21	*loc799704, kcnj8, zgc:194125, kcnb2, slc5a1, slc13a1, slc8a2a, tmem38a, slc13a2, slc40a1, si:dkey-5g7.3, kctd7*
	Muscle organ development	6	2.16E-2	3.73	*myl7, myod1, speg, ndrg4, tnnt2a, lox*
	Cation transport	14	2.97E-2	1.92	*zgc:194125, kcnb2, slc5a1, tmem38a, si:dkey-5g7.3, kctd7, p2rx5, loc799704, kcnj8, slc8a2a, slc13a1, slc13a2, slc30a10, slc40a1*
	Ion transport	17	3.66E-2	1.72	*zgc:194125, kcnb2, slc5a1, tmem38a, si:dkey-5g7.3, kctd7, p2rx5, loc799704, slc26a5, kcnj8, grin1b, slc8a2a, slc13a1, slc13a2, slc30a10, slc34a2a, slc40a1*
	Response to xenobiotic	3	4.05E-2	9.19	*sult1st1, cyp3a65, zgc:174006*
	Response to virus	3	4.61E-2	8.58	*mxe, atf3, mxb*
Cellular component	Endoplasmic reticulum	14	3.54E-3	2.47	*sgk1, creld2, tmem38a, pdia4, hyou1, hsp90b1, slc35b1, loc792835, neu3.1, sdf2l1, pdip5, hspa8, hsd17b12a, neu4*
Molecular function	Nucleotidyltransferase activity	6	3.17E-2	3.38	*pole2, papss2b, si:dkey-57a22.7, uap1l1, pcyt1ba, eif2b3*
	Oxidoreductase activity	3	3.71E-2	9.65	*loxl2a, abp1, lox*
	Dipeptidyl-peptidase activity	2	4.64E-2	45.0	*dpp3, zgc:153024*
KEGG Pathways					
	Steroid hormone biosynthesis	4	1.59E-2	7.32	*cyp3a65, srd5a1, hsd17b12a, hsd17b7*
	Toll-like receptor signaling pathway	6	2.91E-2	3.40	*fos, stat1b, irf7, tlr8a, tlr8b, tlr9*

**Table 2:** Time-dependent expression profiles of genes included in different functional categories in zebrafish liver after exposure to the solvent alone (0.01% DMSO) or 1 mg/L TDCIPP.

Functional categories	Genes	1 day	2 days	4 days
Innate immune	*tlr18*	0.85 ± 0.42	1.93 ± 0.20>*	18.75 ± 6.53*
response	*tlr8a*	0.83 ± 0.23	3.10 ± 0.52*	15.26 ± 5.03*
(GO)/Toll-like	*tlr8b*	0.74 ± 0.27	2.62 ± 0.34*	23.54 ± 10.49*
receptor	*tlr20a*	0.65 ± 0.14	3.67 ± 1.00*	28.73 ± 11.24*
signaling	*tlr9*	0.83 ± 0.23	3.59 ± 1.04*	21.93 ± 9.31*
pathway (KEGG)	*fos*	0.73 ± 0.22	2.62 ± 1.04*	18.14 ± 4.85*
	*stat1b*	1.17 ± 0.38	5.00 ± 1.83*	3.92 ± 1.17*
	*irf7*	1.10 ± 0.30	3.33 ± 1.01*	2.19 ± 0.42*
ER stress	*grp78*	0.96 ± 0.22	1.90 ± 0.24*	10.62 ± 2.88*
	*chop*	1.25 ± 0.27	3.17 ± 0.49*	9.91 ± 3.09*
Inflammation	*il1b*	0.95 ± 0.27	4.50 ± 1.50*	3.40 ± 1.30*
response	*il4*	0.91 ± 0.28	3.75 ± 0.86	0.98 ± 0.47
	*il6*	0.81 ± 0.42	2.48 ± 0.76	12.79 ± 5.20*
	*il10*	0.79 ± 0.30	2.94 ± 0.82	3.56 ± 0.52*
	*il12a*	1.22 ± 0.38	1.44 ± 0.43	5.95 ± 1.23*
	*il13*	0.73 ± 0.33	1.04 ± 0.29	23.24 ± 9.15*
	*il15*	1.01 ± 0.15	1.40 ± 0.18	4.16 ± 1.02*
	*il22*	0.85 ± 0.38	1.37 ± 0.39	5.75 ± 2.46
	*il26*	0.70 ± 0.30	1.19 ± 0.76	23.30 ± 8.81*

Values represent mean ± SEM (n = 9). Significant differences from the control are indicated by *P < 0.05.

**Table 3:** Dose-dependent expression profiles of genes included in different functional categories in zebrafish liver after exposure to the solvent alone (0.01% DMSO) or different concentrations of TDCIPP for 4 days.

Functional categories	Genes	0.1 mg/L	0.3 mg/L	1 mg/L
Innate immune	*tlr18*	0.97 ± 0.28	0.76 ± 0.09	18.75 ± 6.53*
response	*tlr8a*	1.11 ± 0.34	0.70 ± 0.12	15.26 ± 5.03*
(GO)/Toll-like	*tlr8b*	1.18 ± 0.38	0.83 ± 0.21	23.54 ± 10.49*
receptor	*tlr20a*	0.91 ± 0.30	0.78 ± 0.07	28.73 ± 11.24*
signaling	*tlr9*	0.80 ± 0.22	0.63 ± 0.16	21.93 ± 9.31*
pathway (KEGG)	*fos*	1.24 ± 0.49	0.74 ± 0.20	18.14 ± 4.85*
	*stat1b*	6.00 ± 2.00*	4.73 ± 1.63*	3.92 ± 1.17*
	*irf7*	1.34 ± 0.54	2.47 ± 0.67*	2.19 ± 0.42*
ER stress	*grp78*	1.80 ± 0.60	2.08 ± 0.36*	10.62 ± 2.88*
	*chop*	1.21 ± 0.36	0.80 ± 0.13	9.91 ± 3.09*
Inflammation	*il1b*	1.99 ± 0.90	2.10 ± 0.92	3.40 ± 1.30*
response	*il4*	2.76 ± 1.09	1.34 ± 0.40	0.98 ± 0.47
	*il6*	3.77 ± 2.27	2.00 ± 0.90	12.79 ± 5.20*
	*il10*	2.11 ± 1.91	1.74 ± 0.91	3.56 ± 0.52*
	*il12a*	1.19 ± 0.39	0.74 ± 0.24	5.95 ± 1.23*
	*il13*	1.65 ± 0.91	2.35 ± 0.55*	23.24 ± 9.15*
	*il15*	0.64 ± 0.10	0.66 ± 0.21	4.16 ± 1.02*
	*il22*	2.35 ± 0.59	3.15 ± 0.70*	5.75 ± 2.46
	*il26*	1.13 ± 0.75	2.21 ± 1.53	23.30 ± 8.81*

Values represent mean ± SEM (n = 9). Significant differences from the control are indicated by *P < 0.05.
