# Supplementary material for: Acute Exposure to Tris(1,3-dichloro-2-propyl) Phosphate (TDCIPP) Causes Hepatic Inflammation and Leads to Hepatotoxicity in Zebrafish
Source: Sci Rep. 2016 Jan 8;6:19045. doi: 10.1038/srep19045 (PMC4705469; doi:10.1038/srep19045)
Supplement: Supplementary Information [file srep19045-s1.pdf]

**Supporting Information**

# Acute Exposure to Tris(1,3-dichloro-2-propyl) Phosphate (TDCIPP) Causes Hepatic Inflammation and Leads to Hepatotoxicity in Zebrafish


**Chunsheng Liu[1,2,3,*], Guanyong Su[4,5], John P. Giesy[5,6,7], Robert J. Letcher[4], Guangyu Li[1], Ira Agrawal[3], Jing Li[1], Liqin Yu[1], Jianghua Wang[1], Zhiyuan Gong[3,*]**

[1]College of Fisheries, Huazhong Agricultural University, Wuhan 430070, China
[2]Collaborative Innovation Center for Efficient and Health Production of Fisheries in Hunan Province
[3]Department of Biological Sciences, National University of Singapore, Singapore 117543, Singapore
[4]Department of Chemistry, Carleton University, Ottawa, Ontario K1S 5B6, Canada
[5]State Key Laboratory of Pollution Control and Resource Reuse, School of the Environment, Nanjing University, Nanjing 210023, China
[6]Department of Veterinary Biomedical Sciences and Toxicology Centre, University of Saskatchewan, Saskatoon, Saskatchewan S7N 5B3, Canada
[7]Department of Zoology and Centre for Integrative Toxicology, Michigan State University, East Lansing, Michigan 48824, United States

***Authors for correspondence:**

| | |
|---|---|
| Prof. Chunsheng Liu, | Prof. Zhiyuan Gong, |
| College of Fisheries, | Department of Biological Sciences, |
| Huazhong Agricultural University, | National University of Singapore, |
| Wuhan 430070, China | Singapore 117543, Singapore |
| Tel: 86 27 87282113 | Tel: 65162860 |
| Fax: 86 27 87282114 | Fax: 67792486 |
| Email: liuchunshengidid@126.com | Email: dbsgzy@nus.edu.sg |

**Table S1** Sequences of primers for selected genes

| Gene Name | Sequence of the primers (5'-3') | Amplicon size | Accession number |
|---|---|---|---|
| *tlr18* | Forward: ctagagtggcctcagcaacc<br>Reverse: ctcttcatctgggccttcag | 183 bp | NM_001089350.1 |
| *tlr8a* | Forward: tgaggacgtgattgttctgc<br>Reverse: gcctggttgtcgactcgtat | 171 bp | AY389452.1 |
| *tlr8b* | Forward: tgaagagggtgtggatgtga<br>Reverse: caaacctaaccgcgtttctc | 170 bp | AY389453.1 |
| *tlr20a* | Forward: tttcgagaggctttgcgtat<br>Reverse: tgtcgtgtcccaactgaaag | 178 bp | AY389457.1 |
| *tlr9* | Forward: atgcccaaacaaccagtctc<br>Reverse: gtaaaaggtgccgttttgga | 196 bp | NM_001130594.1 |
| *fos* | Forward: gctccatctcagtcccagag<br>Reverse: agagtgggctccagatcaga | 160 bp | NM_205569.1 |
| *stat1b* | Forward: ctccaggcactttccttctg<br>Reverse: cattggagcagcaagtgtgt | 185 bp | NM_200091.2 |
| *irf7* | Forward: gcttcagtccagcaatcaca<br>Reverse: cgtatttgctcccctctcag | 167 bp | NM_200677.1 |
| *gclc* | Forward: aaaatgtccggaactgatcg<br>Reverse: aacgtttccattttcgttgc | 157 bp | NM_199277.2 |
| *gsr* | Forward: caaccttgaaaagggcaaaa<br>Reverse: aaactggatcctggcacatc | 171 bp | NM_001020554.1 |
| *nqo1* | Forward: ctcaaggatttgccttcagc<br>Reverse: cgcagcactccattctgtaa | 169 bp | NM_001204272.1 |
| *chop* | Forward: atatactgggctccgacacg<br>Reverse: ttcgttcttcttgccttggt | 198 bp | NM_001082825.1 |
| *grp78* | Forward: caagaagaagacgggcaaag<br>Reverse: ctcctcaaacttggctctgg | 178 bp | NM_213058.1 |
| *il1b* | Forward: cgctccacatctcgtactca<br>Reverse: atacgcggtgctgataaacc | 166 bp | NM_212844.2 |
| *il4* | Forward: gtgaatgggatcctgaatgg<br>Reverse: ttccagtcccggtatatgct | 190 bp | NM_001170740.1 |
| *il6* | Forward: tcctggtgaacgacatcaaa<br>Reverse: tcatcacgctggagaagttg | 177 bp | NM_001261449.1 |
| *il10* | Forward: atttgtggagggctttcctt<br>Reverse: agagctgttggcagaatggt | 198 bp | NM_001020785.2 |
| *il22* | Forward: cttggaatcagacgagcaca<br>Reverse: ggccaaatccataattgcac | 175 bp | NM_001020792.1 |

**Table S1**-*Continued*

| Gene Name | Sequence of the primers (5'-3') | Amplicon size | Accession number |
|---|---|---|---|
| *il13* | Forward: tcgggttttacgttgaaagg<br>Reverse: atctcctcctcagcctgaca | 197 bp | NM_001199905.1 |
| *il15* | Forward: ccagaacagggactggaaga<br>Reverse: ccctggtgagtcttctcctg | 192 bp | NM_001039565.1 |
| *il12a* | Forward: gaactcctacaagcccagca<br>Reverse: cggatgtgaaacccttcagt | 185 bp | NM_001007107.1 |
| *il26* | Forward: aatgcagaactgtgcgactg<br>Reverse: cctgaactgatccacagcaa | 156 bp | NM_001020799.1 |
| *il34* | Forward: gaacatccacacgcatgaac<br>Reverse: aaaatgaaggagctggctga | 160 bp | NM_001082955.1 |
| *gapdh* | Forward: gatacacggagcaccaggtt<br>Reverse: gccatcaggtcacatacacg | 163 bp | NM_001115114.1 |

Table S2 Number of clean reads and mapped reads, and their percentage (clean reads/clean reads) in control and TDCPP treatment groups.

| | Control-1 | Control-2 | TDCPP-1 | TDCPP-2 |
|---|---|---|---|---|
| Clean reads | 52,451,294 | 52,208,818 | 55,223,302 | 54,749,504 |
| Mapped reads | 35,069,242 | 35,194,969 | 37,665,881 | 37,621,973 |
| Percentage | 66.86% | 67.41% | 62.21% | 68.72% |

**Figure S1**

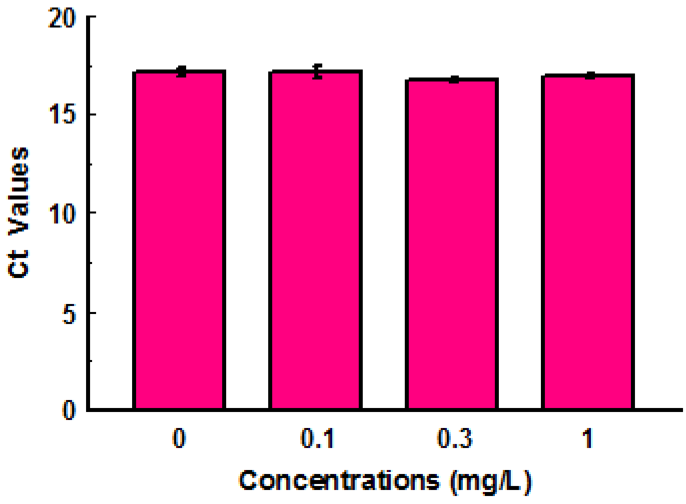


**Figure S1:** Actual cycle threshold (Ct) values for glyceraldehyde-3-phosphate dehydrogenase (*gapdh*) in response to different concentrations of TDCIPP. Values represent mean ± SEM. There were 3 replicated tanks for each concentration, and three fish from each tank were used and thus totally 9 fish were involved in each treatment.

**Figure S2**

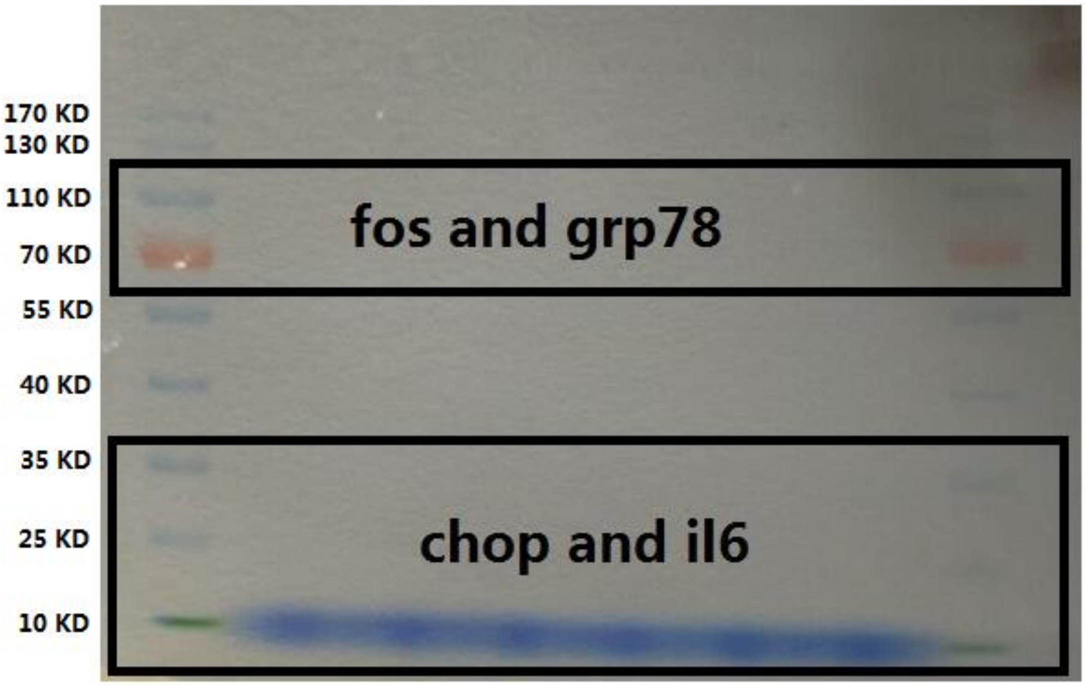


**Figure S2:** Positions where membrane strips were obtained according to molecular weights of marker.

**Figure S3**

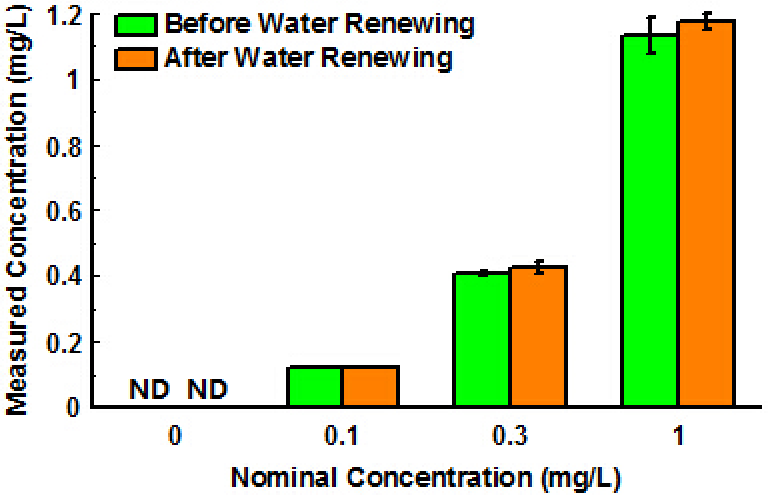


**Figure S3:** Measured concentrations of TDCIPP in exposure solutions at the last day of treatment. Value represent mean±SE (n=3).

**Figure S4**

**(A) Control-1**

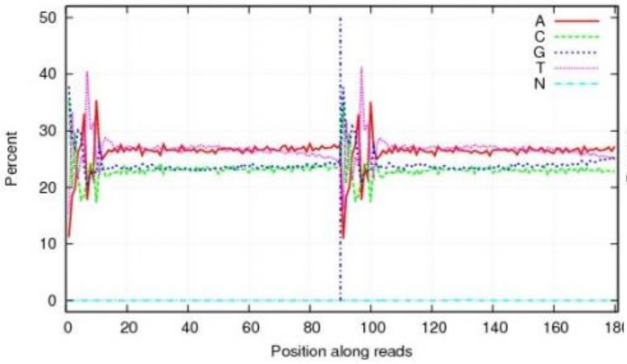


**(B) Control-2**

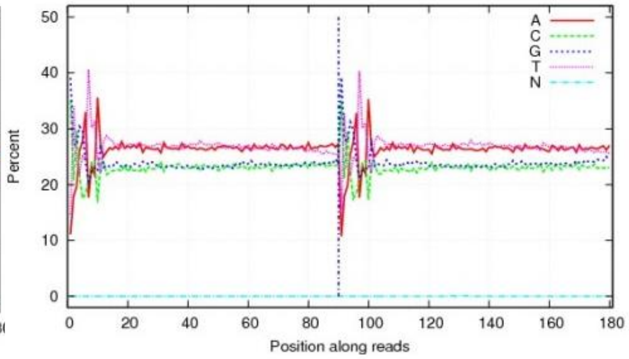


**(C) TDCPP-1**

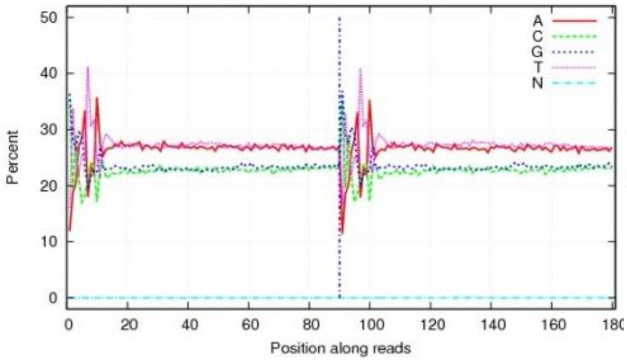


**(D) TDCPP-2**

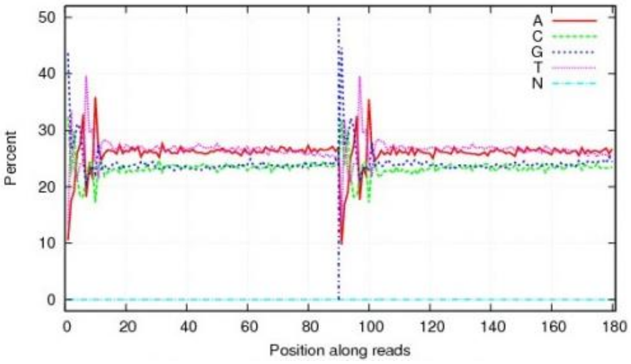


**Figure S4:** Representative images for the composition of bases. T and C curves were in accordance with A and G curves, respectively in all the samples tested, therefore, our results showed satisfactory base composition.

**Figure S5**

**(A) Control-1**

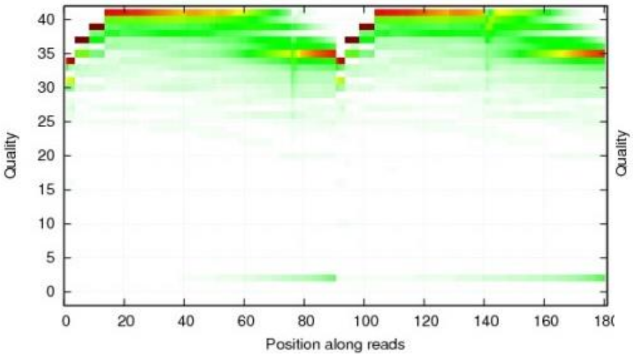


**(B) Control-2**

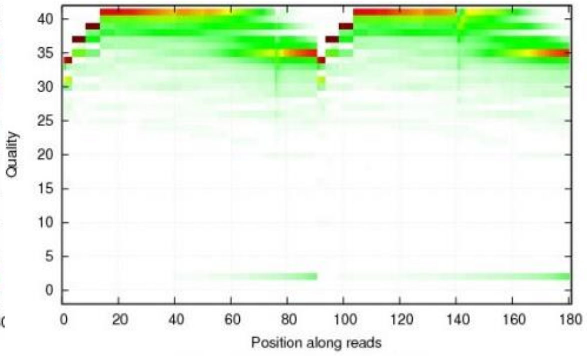


**(C) TDCPP-1**

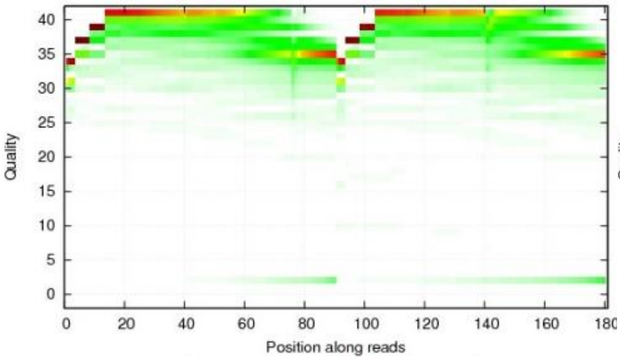


**(D) TDCPP-2**

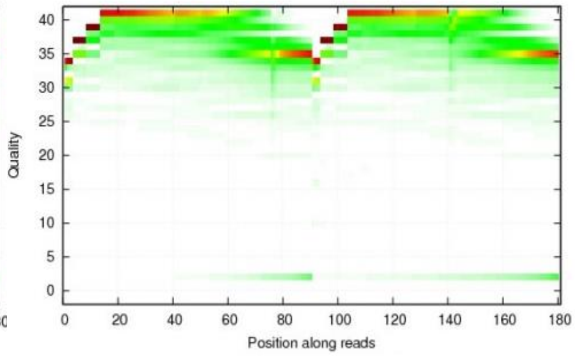


**Figure S5:** Representative images for quality distribution of bases. The percentage of the bases with low quality (<20) was very low in all the samples tested, therefore, the sequencing quality was good in the present study.

**Figure S6**

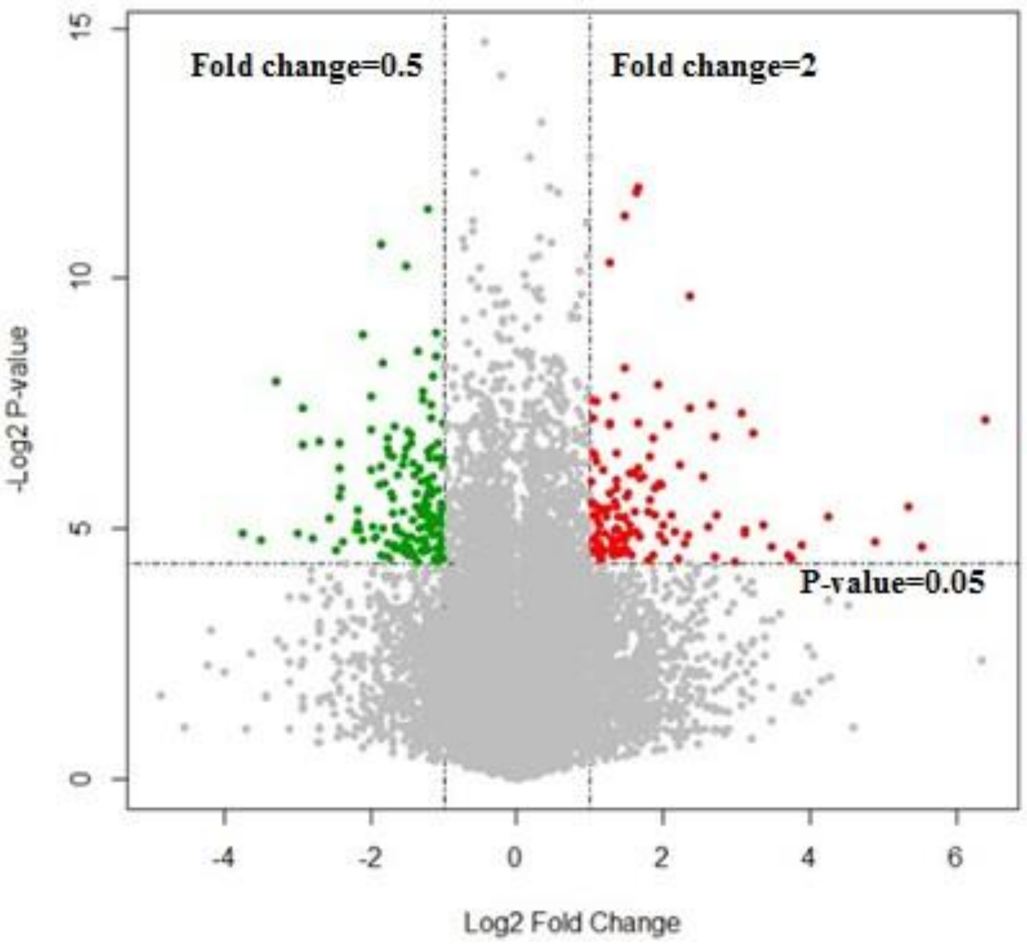


**Figure S6:** Volcano plot showing the distribution of transcripts over different fold change and *P*-value between control and treatment groups. Green plots indicate down-regulated genes, with fold change <0.5 and P-value <0.05; Red plots indicate up-regulated genes, with fold change >2 and P-value <0.05.

**Figure S7**

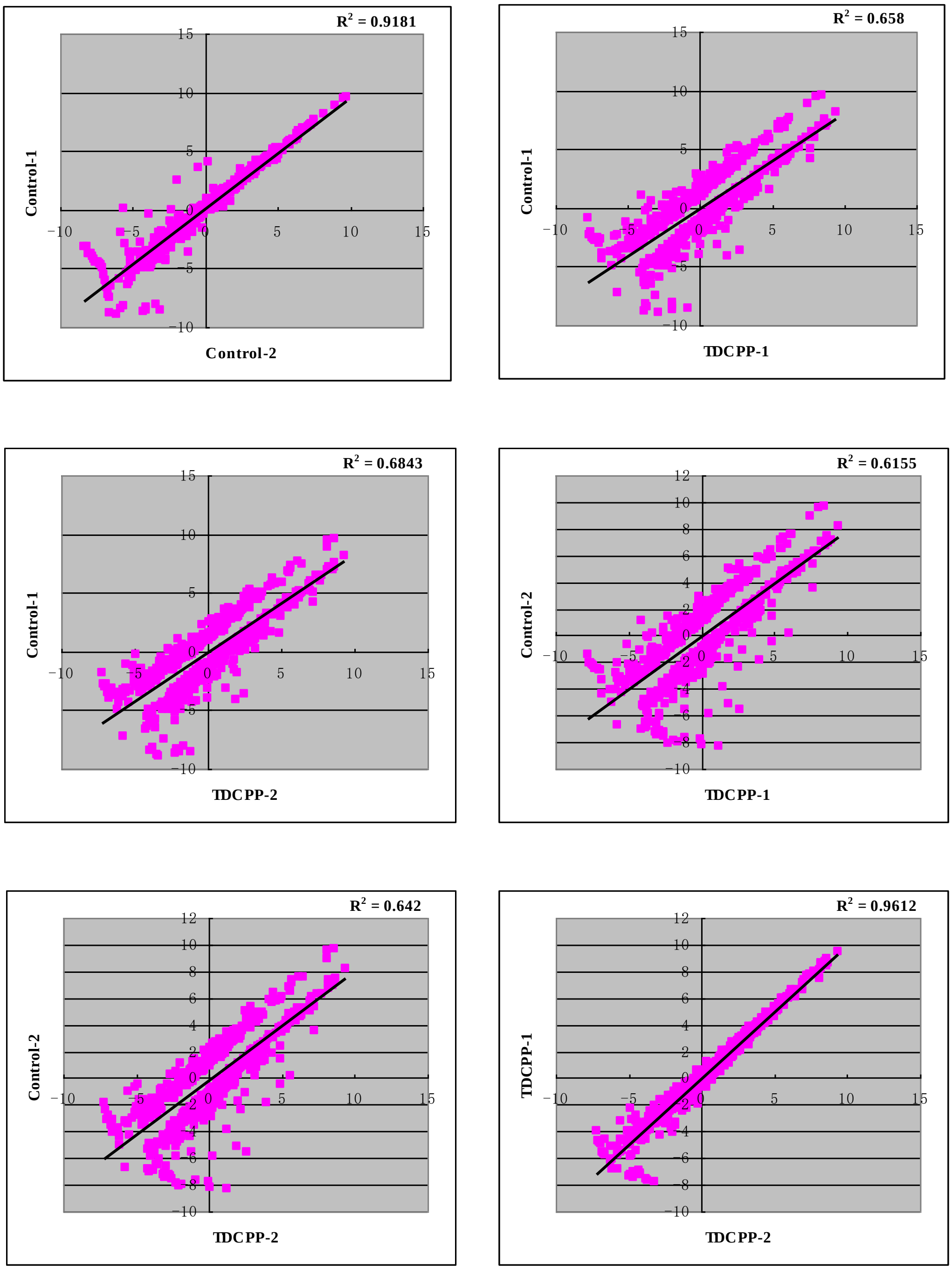


**Figure S7:** Regression analysis between two different RNA-Seq groups based on the relative (log2) RPKM value of identified 583 genes.
